# Morphological Variability of *Pseudo-nitzschia pungens* Clade I (Bacillariophyceae) in the Northwestern Adriatic Sea

**DOI:** 10.3390/plants9111420

**Published:** 2020-10-23

**Authors:** Stefano Accoroni, Sonia Giulietti, Tiziana Romagnoli, Melania Siracusa, Simone Bacchiocchi, Cecilia Totti

**Affiliations:** 1 Dipartimento di Scienze della Vita e dell’Ambiente, Università Politecnica delle Marche, via Brecce Bianche, 60131 Ancona, Italy; s.giulietti@pm.univpm.it (S.G.); t.romagnoli@univpm.it (T.R.); c.totti@univpm.it (C.T.); 2 Istituto Zooprofilattico Sperimentale Umbria e Marche, Via Cupa di Posatora, 3, 60131 Ancona, Italy; m.siracusa@izsum.it (M.S.); s.bacchiocchi@izsum.it (S.B.); 3 Consorzio Interuniversitario per le Scienze del Mare, CoNISMa, ULR Ancona, Ancona, Italy

**Keywords:** domoic acid, ITS, LSU, morphometry, NW Adriatic Sea, phylogeny, ultrastructure, taxonomy, ASP toxins

## Abstract

*Pseudo-nitzschia pungens* is a common component of the phytoplankton community of the northern Adriatic Sea. In this study, an in-depth morphological analysis of *P. pungens* was carried out in both cultured strains isolated in different periods and field samples, revealing a surprisingly wide variability in a number of details, with both the gross morphology and ultrastructural levels deviating from the nominal *P. pungens*. Colonies showed an overlap (from one-third to one-sixth) and a transapical axis (rarely reaching 3 µm), strongly differing from the original description of the species. Moreover, valves may be either symmetrical or slightly asymmetrical, with striae almost always biseriate but sometimes uniseriate or triseriate. Poroids’ morphology in cingular bands was characterized by a wide variability (square, circular, or rectangular poroids without or with up to two hymen sectors), with several combination of them, even within the same cingular band. Phylogenetic analyses based on ITS rDNA showed that the *P. pungens* of the northern Adriatic Sea belonged to clade I. Domoic acid was not detected.

## 1. Introduction

Planktonic diatom species of the genus *Pseudo-nitzschia* are recorded in coastal regions worldwide. Among the 53 known species [1], 26 have been shown to produce domoic acid (DA) [2,3], causing neurologic disorders and memory loss in vertebrates linked to the consumption of contaminated shellfish (Amnesic Shellfish Poisoning) [4,5]. In this genus, the number of genetic lineages is markedly higher than the number of taxa discernible by light microscope (LM). With the description of several new *Pseudo-nitzschia* species, detailing and comparing additional ultrastructural characters has become necessary [6,7,8]. Indeed, several species not easily distinguishable using LM have been described using electron microscopy (EM) coupled with molecular techniques [9,10,11,12,13,14,15,16,17].

Traditionally, species of the genus *Pseudo-nitzschia* have been subdivided in two LM-discernible groups based on cell width in valve view: All the species wider than 3 µm have been combined into the *seriata* group, while those less than 3 µm are in the *delicatissima* group [18]. *Pseudo-nitzschia pungens*, with its varieties, is a cosmopolitan species detected from temperate to tropical waters [8,19,20,21], ascribed to the *seriata* group. The combination of a few morphological characters (including cell size, overlap of cells in colonies [22]) has usually been enough to distinguish *P. pungens* from other *Pseudo-nitzschia* species within the *seriata* group (e.g., *P. fraudulenta* (Cleve) Hasle under LM [23]). Nevertheless, the global distribution of this species and its varieties have received increasing attention after Casteleyn et al. [24,25] recognized the existence of three closely related lineages (i.e., clade I, II, and III) based on ITS rDNA region analyses, which is also supported by morphological and mating studies [8]. These three clades approximately correspond to the morphological varieties *P. pungens* var. *pungens* (clade I), *P. pungens* var. *cingulata* (clade II), and *P. pungens* var. *aveirensis* (clade III) [24,26,27]. To date, among the *P. pungens* varieties, *P. pungens* var. *cingulata* and *P. pungens* var. *aveirensis* are not toxic, while *P. pungens* var. *pungens* is the only one containing both toxic and nontoxic strains [21,27,28,29,30]. There are several morphological features useful to distinguish those clades, such as (i) the shape of the poroids of the valvocopula, (ii) the number of fibulae and striae in 10 µm, (iii) the number of the poroids in 1 µm, (iv) the number of additional poroids in 10 striae, and (v) the transapical axis dimension [8,24]. Moreover, two subgroups of clade III (i.e., IIIa and IIIb) with apparently different geographic distribution (Western Pacific and Western/Northeastern Atlantic strains, respectively) were recently distinguished by ITS sequences [21], although no significant morphological differences have been detected [8]. Regarding the mating studies, an apparent reproductive isolation has been highlighted between clade I and clade III, while strains of clade I and II are sexually compatible and able to produce hybrid offspring with intermediate valve width and structure of valvocopula [31].

*Pseudo-nitzschia pungens* has been commonly recorded in the phytoplankton community of the Mediterranean Sea, although its geographical distribution is seemingly restricted to the northernmost areas of the basin in the NW [28,32,33,34,35], as well as in NE Mediterranean Sea [29], and it is a quite recurrent species in the diatom community of the northern Adriatic Sea [30,36,37,38]. Previous molecular studies carried out on the *P. pungens* from the northern Adriatic have highlighted that the Adriatic strain belonged to clade I [30]. However, an in-depth ultrastructural analysis on the Adriatic *P. pungens* population has not been carried out so far.

Since its first record in 2000 [39], in the Adriatic Sea, the presence of DA in shellfish has been detected only occasionally [7,40], with concentration always well below the EU regulatory limit of 20 mg kg^−1^ [41], despite blooms of several potentially toxic *Pseudo-nitzschia* species such as *P. pungens*, *P. calliantha*, *P. delicatissima*, *P. fraudulenta*, *P. multistriata*, and *P. pseudodelicatissima* [40,42], which commonly occur throughout the year in the study area [43]. High-performance liquid chromatography (HPLC) using UV detection is the most popular method used for the determination of DA in shellfish tissues, which was developed by Quilliam and Wright [44] and modified by AESAN [45]. However, as DA concentrations in *Pseudo-nitzschia* strains and phytoplankton field samples are often very low, more sensitive methods of detection are required. Among other developed methods, LC-MS exhibits good results in terms of sensitivity, accuracy, and selectivity [46], representing one of the best tools to investigate DA presence in cultured strains and phytoplankton field samples.

The aim of this study was to characterize the *P. pungens* population of northwestern Adriatic, based on molecular and ultrastructural features of both natural and culture samples, as well as the analysis of its toxin content.

## 2. Results

### 2.1. Morphology

**Figure 1:**
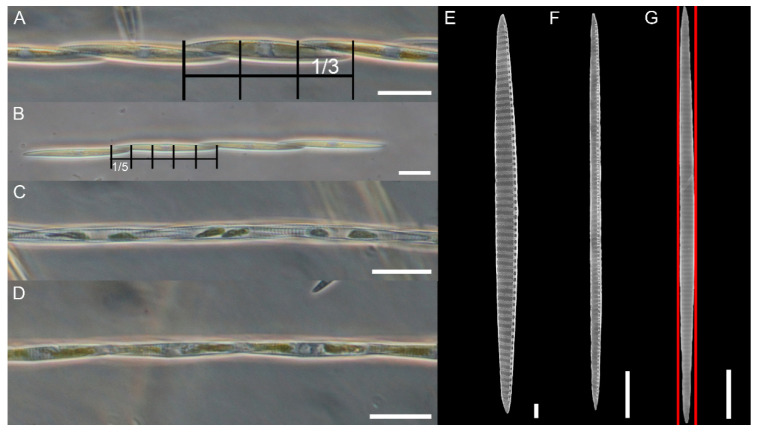
*Pseudo-nitzschia pungens* LM (**A**–**D**) and SEM images (**E**–**G**). (**A**,**B**) Colonies in girdle view, showing a different overlap indicated by the grid (one-third and one-fifth of the total cell length, respectively). (**C**) Colony in valve view. (**D**) Colony in valve view with cells slightly expanding one a side making asymmetrical colony. (**E**–**G**) Valve view of three cells showing the wide Transapical Axis (TA) variability. Red lines mark the asymmetry of the valve. Scale bar = 20 µm (**A**–**D**); 2 µm (**E**); 10 µm (**F**,**G**). Images obtained from the following strains: (**A**,**C**,**D**) 01181; (**B**) 01186; (**E**) 01189; (**F**,**G**) 04196.

**Figure 2:**
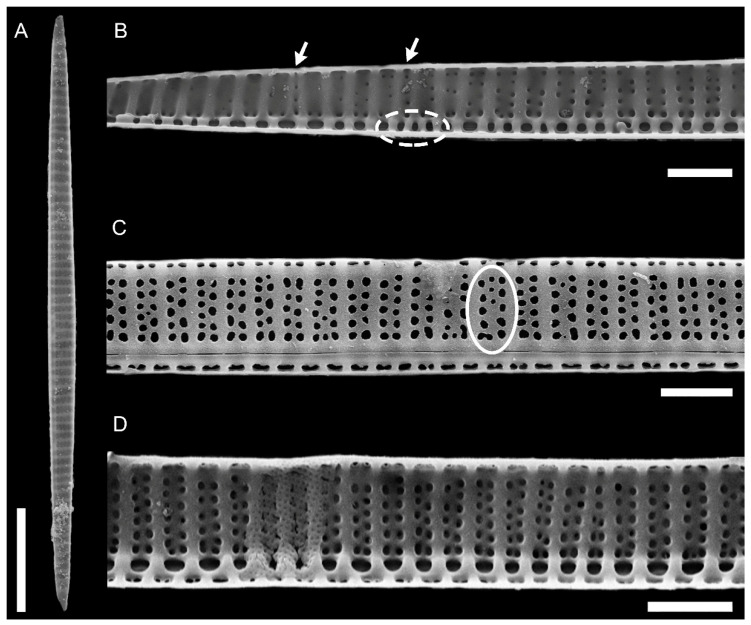
*Pseudo-nitzschia pungens* SEM micrographs of valves. (**A**) Cell in valve view. (**B**) Detail of valve showing the irregular density of fibulae (interrupted circle) and striae (arrows). The decreasing density of poroids toward the apical end is shown. (**C**) Detail of the central part of the valve with an incomplete third row of poroids (white circle). (**D**) Detail of the central part of the valve showing the most common striae pattern with two rows of poroids. Scale bar = 10 µm (**A**); 2 µm (**B**–**D**). Images obtained from field samples.

**Figure 3:**
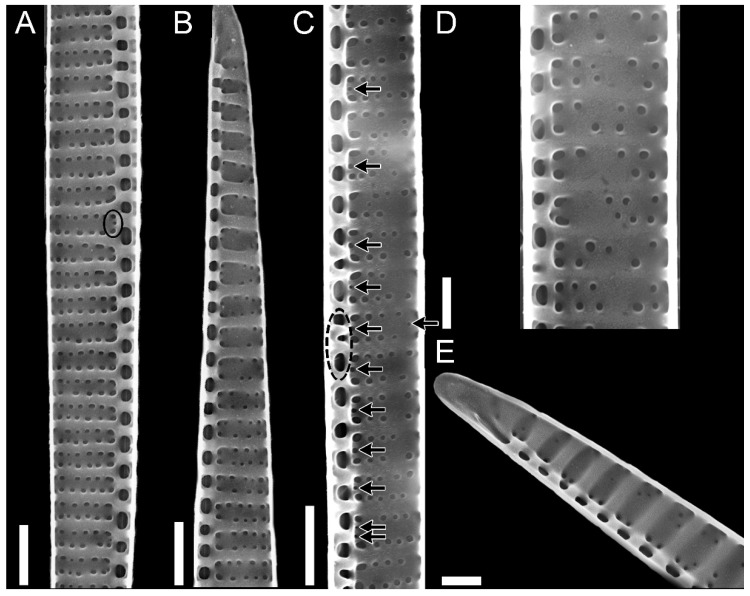
*Pseudo-nitzschia pungens* SEM micrographs of internal valve faces. (**A**) Detail of valve with regular fibulae and striae and an additional poroid (black circle). (**B**) Detail of valve showing lower number of poroids toward the apical part. (**C**) Detail of valve showing irregular density of fibulae (dotted black circle) with several additional poroids (black arrows). (**D**) Detail of the central part of the valve with very low density of poroids. (**E**) Detail of apical part with very low density of poroids. Scale bar = 2 µm (**A**–**C**); 1 µm (**D**,**E**). Images obtained from the following strains: (**A**,**B**) 01186; (**C**,**D**) 04191; (**E**) 01185.

Cells of *Pseudo-nitzschia pungens* from culture material (Figures S1–S9) and from field samples showed very similar morphological and morphometric features. Cells had fusiform to lanceolate shape in both girdle (Figure 1A,B,E–G) and valve view (Figure 1C,D and Figure 2A). All the morphometric measurements have been performed with EM, except for the Apical Axis (AA) measured both in LM and SEM. The AA ranged from 51.1 µm to 127.6 µm, while the Transapical Axis (TA) ranged from 2.0 µm to 3.7 µm (Table 1 and Table S1). The central nodule was absent, and the raphe continued for the full length of the cells (Figure 3A).

Cells formed stepped colonies of several cells with an overlap that ranged from one-third to one-sixth of the AA length (Figure 1A,B, Table 1). Cells were strongly silicified. The interstriae and fibulae were discernible in LM (Figure 1A,C). Although colonies were generally symmetrical in valve view (Figure 1C), asymmetrical colonies were not rare (Figure 1D).

In valve view, valves were slightly asymmetrical with minor hemivalve to major hemivalve area ratios ranging from 0.56 to 0.99 (*n* = 49, 0.82 ± 0.12) and from 0.57 to 0.94 (*n* = 13, 0.80 ± 0.12) in cultured and field samples, respectively. The number of fibulae in 10 µm was 5–18, and the number of striae in 10 µm was 9–16 (Table 1). The number of fibulae was generally the same as the number of striae (Figure 2D and Figure 3A). At times, the number of fibulae was higher (Figure 2B and Figure 3C), but it was rarely lower.

**Figure 4:**
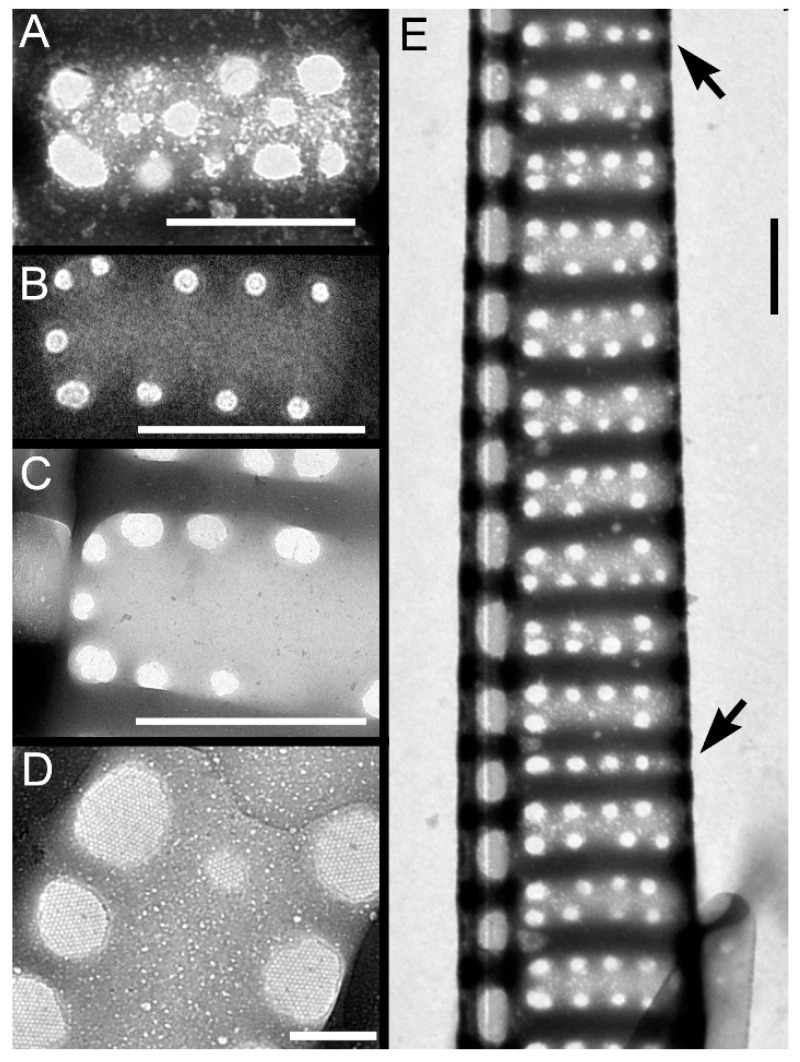
*Pseudo-nitzschia pungens* TEM micrographs of valves. (**A**–**D**) Detail of striae showing (**A**) an incomplete third row of poroids, (**B**–**C**) two additional poroids in the area of the stria close to the raphe, (**D**) additional poroid in the internal part of the stria. (**E**) Detail of valve face with regular density of fibulae and irregular density of striae (arrows indicate striae with only one row of poroids). Scale bar = 1 µm (**A**–**C**,**E**); 0.2 µm (**D**). Images obtained from the following strains: (**A**,**E**) 031832; (**B**,**D**) 01185; (**C**) 01186.

Striae were almost always (72% of the observations) biseriate (Figure 2C,D and Figure 3), with rounded poroids without hymen sectors (Figure 4A–D). Moreover, 3–4 additional poroids in each stria were observed in the 60% of the observations (additional poroids in 10 striae = 2.9 ± 2.79, *n* = 41) (Figure 3C and Figure 4B,C), forming an incomplete third row (Figure 2C and Figure 4A). The presence of additional poroids was more frequent in the part of the stria close to the raphe (Figure 3C and Figure 4B–D). Sometimes (28%), striae were uniseriate (Figure 2B and Figure 4E). The number of poroids in 1 µm ranged between 1 and 4 (Table 1). Cells with a very low density of poroids were not rare (22%, Figure 3D), and a decreasing density of poroids from the valve center toward the apices was often observed (Figure 2B and Figure 3B,E).

**Figure 5:**
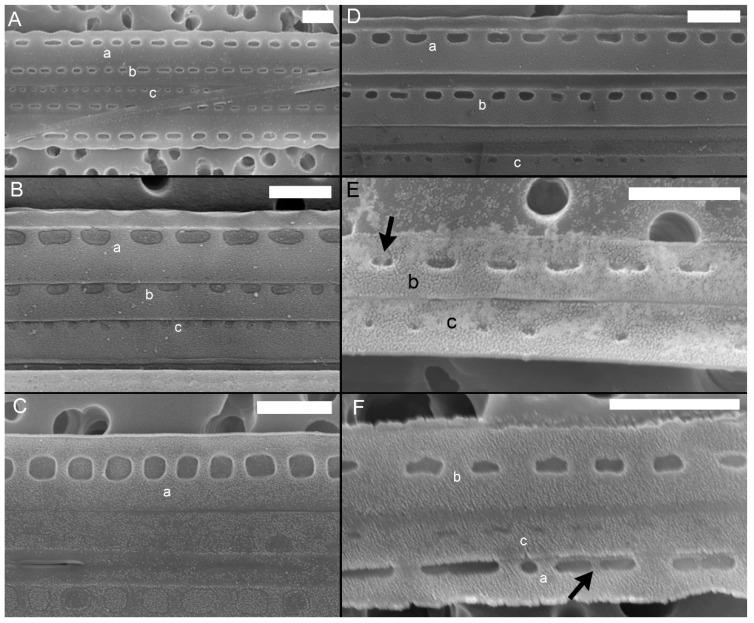
*Pseudo-nitzschia pungens* SEM micrographs of cingular bands: (**a**) Valvocopula; (**b**) second cingular band; (**c**) third cingular band. Band striae was perforated by (**A**,**B**) oval to rectangular, (**C**) square, or (**E**,**F**) circular poroids. (**A**,**D**,**F**) Different types or (**B**,**C**) only one type of poroids could occur in the valvocopula. Black arrows indicate sectors dividing poroids. Scale bar = 1 µm (**A**–**F**). Images obtained from the following strains: (**A**) 04196; (**B**) 01186; (**C**) 01189; (**D**) 04191; (**E**) 04194; (**F**) 05199.

**Figure 6:**
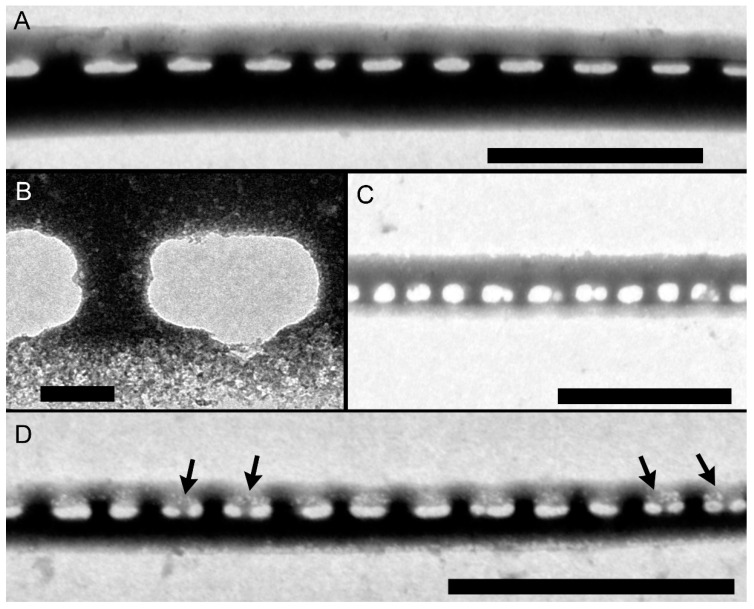
*Pseudo-nitzschia pungens* TEM micrographs of valvocopulae with different poroid pattern: (**A**,**B**) with no sectors, (**C**) one entire or partially divided sector, (**D**) two sectors (black arrows). Scale bar = 2 µm (**A**); 0.2 µm (**B**), 3 µm (**C**,**D**). Images obtained from the following strains: (**A**) 01185; (**B**) 01186; (**C**,**D**) 031832.

In girdle view, cingular bands showed a wide morphological variability both in the shape of poroids and in the number and size of sectors within them (Figure S10A,B, respectively), even within the same cingular band (Figure 5 and Figure 6). In general, poroids’ dimensions showed a decreasing trend in the abvalvar direction (i.e., poroids in the third cingular band were narrower than those in the first two, and sometimes (25%), no poroids were detected), even if, often (40%), the dimensions and the shape of poroids in the first two cingular bands did not differ so much (but the second band had more striae with smaller poroids than the valvocopula, e.g., Figure 5A). Band striae (12–23 in 10 µm, mean 16.2 ± 2.7, *n* = 88) were perforated, with (a) oval to rectangular, (b) square, or (c) circular poroids (Figure S10), showing one, two (partially to completely divided), or no hymen sectors (Figure 6). Within each cingular band, either poroids characterized by only one shape (Figure 5C or Figure 5B(a)) or poroids with different shapes (Figure 5D–F) could occur. Nevertheless, cingular bands with only circular poroids were never observed.

All the patterns of hymenation were observed in poroids (Figure 6), except for circular poroids that were observed only without hymen sectors (Figure 6A) or with one sector.

### 2.2. Molecular Analyses

BLAST results of the 13 LSU sequences confirmed the identification of *Pseudo-nitzschia pungens* (showing from 99 to 100% of identity with *Pseudo-nitzschia pungens* LSU sequences from GenBank).

The final alignment was obtained from a total 94 ITS1-5.8s-ITS2 rDNA sequences of *P. pungens* from different geographical locations, including four sequences from this study.

**Figure 7:**
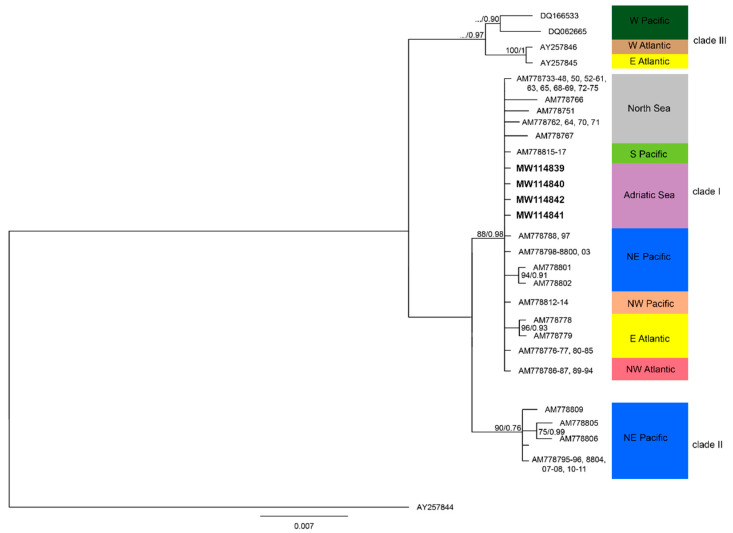
Bayesian consensus tree based on ITS1-5.8s-ITS2 of *Pseudo-nitzschia pungens* rooted with *P. multiseries* (AY257844). Sequences from this study are in bold. Only ML bootstraps values ≥ 70%, and BI posterior probabilities (PP) ≥ 0.90 are shown. (ML/PP). Scale bar = substitutions/site.

The complete alignment was rooted with *P. multiseries* (AY257844). The alignment comprised 632 characters, of which 116 were variable sites and 31 were parsimony-informative. Three clades were recovered (Figure 7).

ML and BI analysis revealed the *P. pungens* strains of this study fell into clade I with a strongly supported bootstrap value (88), and there was a p-distance value of 0.0004 between them and the other sequences of clade I. Clade I was closely related to clade II (mean p-distance value: 0.0143), while the highest p-distance was observed with clade III (mean p-distance value: 0.0319). The highest p-distance among *P. pungens* clades were observed between clade II and clade III (mean p-distance value: 0.0356).

### 2.3. Toxin Content

None of the tested strains by LC-MS/MS produced DA in detectable amounts. The LOD varied between 0.09 and 0.02 fg cell^−1^.

## 3. Discussion

In this study, a significant morphological variability in *Pseudo-nitzschia pungens* populations from the northern Adriatic Sea was highlighted by analyzing a consistent number of samples from both field and cultured material.

The molecular characterization revealed that the Adriatic population belonged to the clade I, as the *P. pungens* nominal variety [24]. However, a number of morphological and ultrastructural details differed from the original description. In the earliest description of *P. pungens*, an overlap of one-third of the cells in colony (or more) and a transapical axis (TA) wider 3 µm has been reported [47], as well as for all the other species belonging to the *seriata* group. Nevertheless, the *P. pungens* from this study revealed an overlap (from one-third to one-sixth) and a TA (rarely reaching 3 µm), strongly differing from the original description of the species, but in agreement with what previously reported for several *P. pungens* strains, irrespective of clade/variety (Table 1). Indeed, the increasing studies focusing on this species have shown that a number of morphological features, previously indicated as key characters, would be not strictly respected. Some authors have already reported an overlap much lower (ranging from one-fourth to one-sixth) in Canada, the Bay of Fundy [53], the northeastern Adriatic Sea [36], the North Sea [54], the Gulf of Mexico [56], the Danish coastal waters [48], and the Atlantic coast of Portugal (*P. pungens* var. *aveirensis*) [27], and a TA often less than 3 µm (down to 1.9), especially in the Mediterranean coast of Greece [29], the northeastern Adriatic Sea [36], and the Atlantic coast of Portugal (*P. pungens* var. *aveirensis*) [27] (Table 1).

Although striae are generally reported as biseriate, an incomplete third row of poroids was very common. Such third row of poroids has been detected in other strains included in all other clades/varieties [24,27,47,48]. On the contrary, the presence of uniseriate striae was reported for the first time in this study.

All the main morphological data of the Adriatic *P. pungens* matched with those of the other clades/varieties, except for (i) the density of band striae in 10 µm (12–23) that slightly diverges from that reported in Pacific coast of USA (California) and in Atlantic coast of Portugal (for *P. pungens* var. *cingulata* and *P. pungens* var. *aveirensis*, 20–24 and 21–25, respectively) [26,27], (ii) the density of fibulae in 10 µm (5–18) that slightly diverges from that reported in Danish coastal waters (10–20) [48], and (iii) the density of poroids in 1 µm (1–4) that slightly diverges from that reported in the Pacific coast of USA (Washington State) (4–5) [51] (Table 1).

In this study, the cell asymmetry of *P. pungens* in valve view has been highlighted for the first time, although it could be noticed looking at the TEM micrographs of *P. pungens* var. *aveirensis* ([27], Figure 47).

Several ultrastructural features have been indicated as useful to discriminate among *P. pungens* varieties, such as the shape and pattern of poroids in the cingular bands (Figure S11) [24,27]. While the nominal variety (*P. pungens* var. *pungens*) has three cingular bands, all with one row of oval to rectangular poroids [22], *P. pungens* var. *cingulata* is characterized by different cingular bands, i.e., the valvocopula has square poroids having two rows of 2–3 hymen sectors, while the second band has rectangular poroids characterized by 1–2 hymen sectors; no descriptions have been reported for the third band [26]. A further poroids’ pattern was described by Churro et al. [27] who established the variety *P. pungens* var. *aveirensis*, having two types of cingular bands, valvocopula with square poroids split into two to three parts and the second band with one row of oval (sometimes split) poroids ([27], Figure 51 and Figure 51 insert).

The morphology of the poroids in cingular bands of the Adriatic *P. pungens* was characterized by a wide variability among strains and within strains and among field samples, showing a number of different combinations of ultrastructural details previously used to discriminate the *P. pungens* varieties described so far. In fact, cingular bands could have square, circular, or rectangular poroids without or with 1–2 hymen sectors, with several combination of them, even within the same cingular band. As a consequence, this high variability in ultrastructural detail patterns makes such details uninformative for discriminating the N Adriatic *P. pungens* (belonging to clade I, as the *P. pungens* nominal variety [24]) from *P. pungens* var. *aveirensis.* On the contrary, N Adriatic *P. pungens* clearly differs from *P. pungens* var. *cingulata*.

Poroids’ dimensions in the cingular bands of *P. pungens* var. *cingulata* and *P. pungens* var. *aveirensis* showed a decreasing trend in the abvalvar direction (i.e., poroids in the third cingular band were narrower than those in the first two cingular bands) [26,27], as often observed in this study for *P. pungens* from the Adriatic Sea. However, differently from the other varieties, in our samples, it was not rare to observe that, in the dimensions of poroids, the first two cingular bands did not differ, so much that valvocopula and the second cingular band were not always easily discernable. In these cases, the second cingular band could be distinguished because of the slightly higher number of band striae in 10 µm.

Until now, the *P. pungens* varieties have been approximatively ascribed to the three clades: *P. pungens* var. *pungens*~clade I, *P. pungens* var. *cingulata*~clade II, and *P. pungens* var. *aveirensis*~clade III [24,26,27]. Nevertheless, results of this study suggest that a clade does not necessary correspond to a morphological variety and vice versa. Indeed, Adriatic *P. pungens* clade I showed a wide morphological variability, covering at least two varieties (i.e., *P. pungens* var. *pungens* and *P. pungens* var *aveirensis*).

Field and experimental studies showed that diatom frustules can be significantly modified by environmental conditions such that genetically identical individuals could be identified as different species [58,59]. For example, salinity and temperature, among other conditions, have strong effects on frustule morphology, clearly demonstrating the flexibility in diatom morphogenesis [58,60,61]. Some of the morphological features, defined as key characteristics for diatom taxonomic identification, have been shown to be more variable than previously thought [62,63]. In this regard, morphological variability should be investigated under different environmental conditions and in the highest possible number of individuals in order to cover the entire morphological variability within the same population.

The wide morphological and morphometrical variability observed in the *P. pungens* clade I population from Adriatic Sea, often overlapping characteristics proper of different varieties, could be explained by taking in account the great number of observations that were performed in this study compared with the previous ones (Table 1). Moreover, the analyses were conducted under a wide spectrum of conditions (i.e., from field and cultured samples sampled in different periods and with different strains of different ages). Nevertheless, this wide variability was detected also between different specimens belonging to the same sample, suggesting that this variability was intrinsic and only partially ascribable to the different environmental conditions.

Finally, although some strains of *P. pungens* clade I were recorded to be toxigenic [29], none of the cultured strains of *P. pungens* clade I from this study produced DA in detectable amounts, in accordance with what previously observed in NW Adriatic strains [30] and with the results from the official monitoring of shellfish production sites [64] in the NW Adriatic Sea, that only sporadically revealed the presence of DA in shellfish and at very low levels [7,40].

## 4. Materials and Methods

### 4.1. Study Area and Sampling

The study area is the coastal station SG01 (43°45.86′ N, 13°13.00′ E) of the Senigallia-Susak transect located in the southern part of the northern Adriatic subbasin, 1.2 nM from the Italian coastline (bottom depth: 12 m), and is included in the LTER (Long-Term Ecological Research) Italian sites, where phytoplankton and environmental parameters have been sampled since 1988.

Sampling was carried out with at about a monthly frequency, from January 2018 to December 2019. Water samples were collected at surface by Niskin bottles, in 250 mL dark glass bottles and preserved by adding 0.8% formaldehyde prefiltered and neutralized with hexamethylenetetramine [65], and stored at 4 °C until analysis was performed. Moreover, net (20 µm mesh) samples were collected for cell isolation (see below).

### 4.2. Pseudo-Nitzschia Strain Isolation

The isolation of single cells of *Pseudo-nitzschia pungens* was carried out in 24-well plates following the capillary pipette method [66]. Cultures were maintained at 21 °C with a 12:12 h of light:dark photoperiod and an irradiance of 100 µmol m^−^^2^ s^−1^, in sterile filtered seawater enriched with f/2 nutrients [67]. Every month, the algal cultures were checked for their purity and quality and refreshed with fresh culture medium. A total of 14 *Pseudo-nitzschia pungens* strains were set up.

### 4.3. DNA Extraction from Algal Culture PCR Amplification and Sequencing

Of the total 14 strains set up, 13 were used for the molecular analyses. Algal cultures were harvested during their late exponential phase and centrifuged at 4000× *g* for 15 min in order to obtain the pellets. Pellets were extracted using CTAB (*N-cetyl-N,N,N*-trimethylammoniumbromide) buffer (2% CTAB, 1 M Tris pH 8.0, 0.5 M EDTA pH 8.0, 5M NaCl, 1%) modified from Doyle and Doyle [68].

Extracted DNA was amplified by Polymerase Chain Reaction (PCR) technique, carried out with a SimpliAmp^TM^ Thermal Cycler.

The D1-D3 region was amplified using universal primers: forward primer D1R (5′-ACC CGC TGA ATT TAA GCA TA-3′) and reverse primer D3Ca (5′-ACG AAC GAT TTG CAG GTC AG-3′) [69]. The ITS region was amplified using ITS1 (5′-TCC GTA GGT GAA CCT GCG G-3′) and ITS4 (5′-TCC TCC GCT TAT TGA TAT GC-3′) [70].

PCR products were visualized with UV from an agarose gel (1%).

The PCR conditions for LSU and ITS region were 94 °C for 4 min, followed by 35 cycles of 94 °C for 30 sec, annealing at 60 and 58 °C (for LSU and ITS regions, respectively) for 45 sec, and elongation at 72 °C for 1 min, followed by further elongation at 72 °C for 5 min.

### 4.4. Sequence Analyses

Taxonomic assignation was performed by blasting each LSU sequences against the GenBank database (NCBI on-line BLAST web interface version 2.9.0+ [71]) to determine the closest known sequences.

Sequences were adjusted for the presence of double peaks by eye with BioEdit [72]. Among the ITS sequences from this study, 4 were aligned with 90 sequences retrieved from GenBank. *Pseudo-nitzschia multiseries* was the outgroup sequences (Table S2). The selection of outgroup sequences was based on the findings by Lim et al. [21].

Alignments were made with ClustalW [73] using the default setting and were then edited manually. Regions that did not fit with the others were excluded from the phylogenetic analyses. Two independent analyses were used to conduct the ITS1-5.8s-IT2 phylogeny: Maximum Likelihood (ML) and Bayesian Inference (BI). The best nucleotide substitution model was tested with Partitionfinder 2 [74]. The generalized time-reversible evolution model (GTR+G) was used for the construction of the RAxML phylogenetic analysis, and Kimura’s two-parameter model (K80 + I) was used for the Bayesian inference tree. ML analyses were carried out with RAxML [75] 1000 pseudo replicates through Cipress portal [76].

Bayesian analyses were carried out using MrBayes 3.2 [77] with 3,000,000 Markov chain and Monte Carlo generations, a sample frequency of 1500, and a diagnosing frequency of 1000. The 50% majority rule consensus tree was constructed discarding the first 25% of samples. Posterior probabilities were calculated to measure tree strength.

The distance estimation matrix between groups was calculated with the p-distance method using the default setting of MEGA 7 [78].

### 4.5. Morphological Characterization

#### 4.5.1. Light Microscopy Analyses

*Pseudo-nitzschia* cells were measured at 1000× magnification using an inverted microscope (ZEISS Axiovert 135) equipped with phase contrast. The Apical Axis (AA) and the overlapping region of the cells in a chain were measured in at least 100 cells from cultured strains and field samples.

#### 4.5.2. Ultrastructural Characterization (TEM and SEM)

Samples for TEM and SEM analyses were harvested from cultured strains in exponential growth phase, collected during 2018–2019 (Table S3) and from field net samples collected in February and May 2019.

Samples were acid-cleaned following von Stosch’s protocol [18]. A drop (2 µL) of the cleaned material was placed on a grid and on a stub and observed with a Philips TEM 400 microscope and a SEM (FE-SEM; Zeiss Supra 40, Carl Zeiss AG, Oberkochen, Germany), respectively.

Several cells were measured (see Table 1 for the number of cells used for each measurement) both from cultured and field samples for Transapical Axis (TA), fibulae, striae, and poroids’ density in both valves and cingular bands with particular focus to valvocopula.

A measurement of valval symmetry was performed on SEM micrographs, calculating the cell surface with an image analysis software and using the formula as follows: Cells in valval view were divided into two hemivalves by the apical axis, crossing the half of the transapical axis. Then, the symmetry was expressed as the ratio of the two hemivalve areas (minor hemivalve:major hemivalve, Figure S12). Valves were asymmetric when the ratio ≠ 1.

### 4.6. Toxin Content

#### 4.6.1. Chemicals and Standards

The acetonitrile (MeACN) and formic acid (FA) were of LC-MS grade, and the methanol (MeOH) was of HPLC grade. Water was distilled and passed through a MilliQ water purification system (DIW) (Millipore Ltd., Bedford, MA, USA).

Certified reference material for DA, CRM-DA-g (103.3 µg mL^−1^), was purchased from the Institute of Biotoxin Metrology at the National Research Council of Canada (NRCC, Halifax, Nova Scotia, Canada). Calibration solutions of DA were prepared from serial dilutions of the reference material in DIW.

#### 4.6.2. DA Extraction

Chemical analysis of *Pseudo-nitzschia pungens* needs a large quantity of cells, so each strain was grown in an increasing volume up to 2 L to achieve abundances, ranging from 17 × 10^4^ to 61 × 10^4^ cells mL^−1^ among the cultured strains.

The strains were grown in the same culture conditions reported above. Cells were harvested from the early stationary growth phase. Algal pellets of 4 *P. pungens* strains (Table S3) were extracted using a mixture of MeOH/H_2_O (50:50 *v*/*v*), following the official EU-RL RP-LC-UV method (EURLMB 2008), for the determination of DA in shellfish and finfish.

All culture volume (2 L) was centrifuged for 20 min at 2500× *g* (4 °C) in 40 centrifuge tubes (50 mL volume). Pellets were combined and extracted with 5 mL of MeOH/H_2_O (50:50 *v*/*v*), vortex-mixed for 1 min, and bath-sonicated for 10 min. After sonication, the aliquot was centrifuged for 10 min at 2500× *g* (4 °C), and the supernatant was transferred to a 100 mL evaporation flask. Pellet extraction was repeated three times, and the supernatants were combined and evaporated to dryness. The residue was reconstituted in 1 mL of MeOH/H_2_O (50:50 *v*/*v*) and filtered through a 0.2 µm syringe filter (Minisart, Sartorius, Germany) for LC-MS/MS analysis.

#### 4.6.3. LC-MS/MS Analysis

LC-MS/MS analyses were performed using a hybrid triple-quadrupole/linear ion trap 3200 QTRAP mass spectrometer (AB Sciex, Darmstadt, Germany) equipped with a Turbo V source and an electrospray ionization (ESI) probe. The mass spectrometer was coupled to an Agilent model 1200 LC instrument (Palo Alto, CA, USA), which included a solvent reservoir, inline degasser, quaternary pump, refrigerated autosampler, and column oven.

The method was implemented following the conditions described by Mafra et al. [46], which were properly modified. LC separation was performed using a Gemini^®®^ NX-C18 column (2 mm × 100 mm, 3 µm particle size; Phenomenex, Torrance, CA, USA), set at 40 °C, with a flowrate of 0.4 mL min^−1^. Mobile phase A was DIW and B MeACN, both containing 0.2% of FA. Gradient elution was adopted, as described below: From 10% to 20% B in 5 min, from 20% to 35% B in 1 min, then hold for 6 min, return to the original conditions at 13 min, and hold for 7 min before the next injection.

Infusion experiments were performed using CRM-DA-g to set the turbo IonSpray source parameters as follows: Nebulizer Gas (GS1) 50 psi, Auxiliary Gas (GS2) 60 psi, Temperature (TEM) 600 °C, Ion Spray Voltage (IS) 5000 V, Curtain Gas (CUR) 20 psi.

DA was detected using Multiple Reaction Monitoring (MRM) in positive ion mode by selecting the following transitions: *m*/*z* 312.2→266.1, *m*/*z* 312.2→220.1, and *m*/*z* 312.2→161.1. In addition, the pseudotransition *m*/*z* 334.2→334.2 of sodium adduct [DA + Na]^+^ was monitored to investigate ion suppression due to salts. A declustering potential (DP) of 60 V and a collision energy (CE) of 30 V were used for all transitions.

LOQ, calculated assuming a signal/noise (S/N) ratio of 10 was 10 ng mL^−1^, while LOD (S/N ratio of 3) was 3 ng mL^−1^.

## Figures and Tables

**Table 1:** Morphometric characteristics of *Pseudo-nitzschia pungens* reported in literature and in this study. n.r., not reported.

Apical Axis (µm)	Transapical Axis (µm)	Fibulae in 10 µm	Striae in 10 µm	Poroids in 1 µm	Band Atriae (in 10 µm)	Apical Axis/Overlap	Location	Type of Samples	Clade/Variety	References
74–142	2.9–4.5	9–15	9–15	3–4	n.r.	3	n.r.		n.r.	[47]
71–140	2.8–4.5	10–14	10–14	3–4.5	20–24	n.r.	Pacific coast of USA (California)	Field and culture samples	II/cingulata	[26]
100–155 (116.1 ± 13.2) *n* = 74	1.8–4.0 (2.9 ± 0.5) *n* = 60	10–20 (12.3 ± 1.6) *n* = 78	10–14 (12.0 ± 1.1) *n* = 82	2–4 (3.3 ± 0.4) *n* = 81	n.r.	3–5 (4) *n* = 70	Danish coastal waters	n.r.	n.r.	[48]
72–135 (99.1 ± 21.5)	2.4–4.5 (3.9 ± 0.8)	9–13 (10.8 ± 1.9)	9–13 (10.8 ± 1.6)	3–4 (3.4 ± 0.3)	17–18 (17.5 ± 0.4)	n.r.	Sea of Japan	Field samples	n.r.	[49,50]
94–160	2–4	10–16	10–16	4–5	n.r.	n.r.	Pacific coast of USA (Washington State)	Field samples	n.r.	[51]
74–174	2.4–5.3	9–16	9–16	n.r.	n.r.	3	Gulf of Mexico	Field samples	n.r.	[52]
92–156 (113 ± 31) *n* = 3	3.5–4.2 (4.0 ± 0.3) *n* = 3	10–11 (11 ± 0.3) *n* = 4	10–11 (10.5 ± 0.5) *n* = 5	1–3 (2.5 ± 0.6) *n* = 6		4	Canada, Bay of Fundy	Field samples	n.r.	[53]
24.4–121.0 (79.1 ± 8.7) *n* = 70	2.4–3.8 (3.2 ± 0.4) *n* = 70	9–13 (11.9 ± 0.3) *n* = 70	10–14 (11.1 ± 0.5) *n* = 70	2–4 (3.0 ± 0.5) *n* = 50	n.r.	n.r.	North Sea	Culture samples	I/*pungens*	[24]
87.9–108.7 (101.5 ± 27.2) *n* = 42	3.4–4.7 (3.9 ± 0.1) *n* = 42	11–15 (12.7 ± 0.2) *n* = 50	10–13 (11.6 ± 0.3) *n* = 42	3–5 (4.2 ± 0.5) *n* = 50	n.r.	n.r.	North Sea	Culture samples	II/*cingulata*	[24]
74–147 (112 ± 17.6) *n* = 35	2.6–4.5 (3.4 ± 0.5) *n* = 35	10–13 (11 ± 1.2) *n* = 5	9–13 (11.4 ± 1.5) *n* = 5	2.5–3 (2.9 ± 0.2) *n* = 5	n.r.	n.r.	Catalonia, NW Mediterranean	Field samples	n.r.	[34]
70–156 116.9 ± 24.6 *n* = 81	2.2–4.8 (3.75 ± 0.57) *n* = 81	9–13 (11.2 ± 1.3) *n* = 10	9–13 (11.4 ± 1.4) *n* = 10	2.5–3 (3.0 ± 0.2) *n* = 10	n.r.	n.r.	Catalonia, NW Mediterranean	Field samples	n.r.	[35]
86.3–160.8 (104.6 ± 10.42) *n* = 50	3.7–5.3 (4.5 ± 0.35) *n* = 50	10–16 (12.8 ± 1.4) *n* = 50	10–13 (11.1 ± 0.68) *n* = 50	n.r.	n.r.	3–5	North Sea	Field samples	n.r.	[54]
47–100 (67.7 ± 14.8)	2.7–3.7 (3.3 ± 0.6)	13–16 (14.8 ± 0.7)	13–16 (14.9 ± 0.7)	3–5 (4.0 ± 0.0)	21–25 (23.0 ± 1.1)	5–6	Atlantic Ocean, Portugal	Culture samples	III/ *aveirensis*	[27]
84–165	3.0–5.0	13–18	Striae n.r. Interstriae: 13–16	2–3	n.r.	n.r.	Atlantic Ocean, southern Brazil	Field samples	*pungens*	[55]
89–122	3.0–4.0	13–14	Striae n.r. Interstriae: 12–14	3–4	n.r.	n.r.	Atlantic Ocean, southern Brazil	Field samples	*cingulata*	[55]
n.r.	1.9–3.2 (2.5 ± 0.41) *n* = 28	10–17 (13 ± 2.3) *n* = 8	8–15 (13 ± 2.3) *n* = 8	2–4 (3 ± 0.6) *n* = 8	n.r.	4.5–4.8	NE Adriatic Sea	Field samples	n.r.	[36]
n.r.	2.5–3.6 (2.9 ± 0.3) *n* = 75	11–14 (12.2 ± 0.9) *n* = 71	11–14 (12.0 ± 0.8) *n* = 71	2–4 (3.5 ± 0.6) *n* = 105	14–20 (16.5 ± 1.6) *n* = 45	n.r.	Northern Aegean Sea	Culture samples	I	[29]
93–126	2.8–3.2	11–15	11–14	3–3.4	n.r.	3–4	Gulf of Mexico	Field samples	n.r.	[56]
72–149	3.0–4.1	11–13	10–12	3–4	15–23	n.r.	Atlantic Ocean, Gulf of Maine	Culture samples	n.r.	[57]
80–92 (86.21 ± 3.66) *n* = 24	2.4–4.2 (3.75 ± 0.96) *n* = 24	9–13 (11.4 ± 1.2) *n* = 10	10–13 (11.2 ± 0.9) *n* = 10	2–4 (2.97 ± 0.45) *n* = 35	n.r.	n.r.	NE Adriatic Sea (Gulf of Trieste)	Culture samples	I	[38]
51.1–99.4 (78.9 ± 11.7) *n* = 213	2.0–3.6 (2.82 ± 0.32) *n* = 80	5–18 (11.6 ± 1.9) *n* = 79	9–16 (11.3 ± 1.2) *n* = 81	1–4 (3 ± 0.6) *n* = 90	12–23 (16.2 ± 2.7) *n* = 88	2.9–6.0 (3.9 ± 0.6) *n* = 130	NW Adriatic Sea (Senigallia LTER station)	Culture samples	I	this study
57.2–127.6 (93.9 ± 25.8) *n* = 26	2.4–3.7 (2.8 ± 0.33) *n* = 15	9–16 (11.6 ± 1.98) *n* = 13	9–14 (11.3 ± 1.3) *n* = 14	3–4 (3.4 ± 0.5) *n* = 15	n.r.	3.7–5.2 (4.6 ± 0.5) *n* = 14	NW Adriatic Sea (Senigallia LTER station)	Field samples	I	this study

## Supplementary Materials

The following are available online at https://www.mdpi.com/2223-7747/9/11/1420/s1, Figure S1: *Pseudo-nitzschia pungens* SEM micrographs (Strain ID = 01185). (A) Valve view. (B) Detail of the biseriate striae with low density of poroids. (C) Girdle view. Scale bar = 2 µm, Figure S2: *Pseudo-nitzschia pungens* SEM micrographs (Strain ID = 01186). (A) Valve view. (B) Detail of the biseriate striae. (C) Girdle view: dimensions and shape of poroids in the first two cingular bands are similar. Scale bar = 2 µm, Figure S3: *Pseudo-nitzschia pungens* SEM micrographs (Strain ID = 01189). (A) Valve view. (B) Cingular bands showing square and rectangular poroids. Scale bar = 2 µm, Figure S4: *Pseudo-nitzschia pungens* SEM micrographs (Strain ID = 031832). (A–C) Valve view showing different patterns of symmetry (highlighted by the white lines). Scale bar = 10 µm, Figure S5: *Pseudo-nitzschia pungens* SEM micrographs (Strain ID = 04191). (A) Valve view. (B, C) Detail of the biseriate striae with different density of poroids. (D) Girdle view: dimensions and shape of poroids in the first two cingular bands are similar, while the third cingular band has smaller poroids. Scale bar = 10 µm (A); 2 µm (B, D); 1 µm (C), Figure S6: *Pseudo-nitzschia pungens* SEM micrographs (Strain ID = 04194). (A) Valve view. (B) Detail of the biseriate striae. (C, D) Girdle view: (C) dimensions and shape of poroids in the first two cingular bands are similar; (D) poroids with different shapes and hymenation occurring in the same cingular band. Scale bar = 10 µm (A); 2 µm (B–D), Figure S7: *Pseudo-nitzschia pungens* SEM micrographs (Strain ID = 04196). (A) Valve view of the internal valve face. (B) Detail of the biseriate striae with low density of poroids. (C) Girdle view showing a decreasing trend in abvalvar direction of the poroids’ dimensions. Scale bar = 10 µm (A); 2 µm (B–C), Figure S8: *Pseudo-nitzschia pungens* SEM micrographs (Strain ID = 05197). (A) Valve view. (B) Detail of the biseriate striae. (C, D) Girdle view: (C) dimensions and shape of poroids in the first two cingular bands are similar, while the third cingular band has smaller poroids; (D) poroids with different hymenation occurring in the same cingular band. Scale bar = 10 µm (A); 1 µm (B, D); 2 µm (C), Figure S9: *Pseudo-nitzschia pungens* SEM micrographs (Strain ID = 05199). (A) Valve view. (B) Detail of the biseriate striae. (C, D) Girdle view: (C) dimensions and shape of poroids in the first two cingular bands are similar; (D) poroids with different shape and hymenation occurring in the same cingular band. Scale bar = 10 µm (A); 2 µm (B, C); 0.3 µm (C), Figure S10: Schematic drawing describing the poroids’ morphologies of N Adriatic Sea *Pseudo-nitzschia pungens*. (A) Shapes of poroids: Type a: oval to rectangular. Type b: square poroids. Type c: circular. (B) Pattern of hymenation: Type I: no hymen sectors. Type II: one hymen sectors. Type III: two hymen sectors. Type IV: one partially divided hymen sector, Figure S11: Drawing representing the ultrastructure of poroids in the cingular bands of the three *P. pungens* varieties: (A) var. *pungens* [22]; (B) var. *cingulata* [26]; (C) var. *aveirensis* [27]. VC: valvocopula; II: second cingular band; III: third cingular band. No information about the third cingular band is available in var. *cingulata* and var. *aveirensis*, Figure S12: *Pseudo-nitzschia pungens* cell divided into two hemivalves. Green area: minor hemivalve; red area: major hemivalve, Table S1: Morphometric characteristics of *Pseudo-nitzschia pungens* strains from the coastal site SG1 of LTER Senigallia transect. n.r., not reported, Table S2: List of sequences retrieved from Genbank for the construction of the Bayesian consensus tree. Strains from this study are in bold. (c) strains of which PCR products were cloned, Table S3: List of *Pseudo-nitzschia pungens* strains from the coastal site SG1 of LTER Senigallia transect, cleaned for morphological characterization with TEM and SEM. Strain IDs indicated in bold are those analyzed for toxin content.
