# Supplementary material for: Morphological Variability of Pseudo-nitzschia pungens Clade I (Bacillariophyceae) in the Northwestern Adriatic Sea
Source: Plants (Basel). 2020 Oct 23;9(11):1420. doi: 10.3390/plants9111420 (PMC7716229; doi:10.3390/plants9111420)
Supplement: Supplementary file 1 [file plants-09-01420-s001.zip › plants-952561-supplementary.docx]

Supplementary Materials

Morphological variability of *Pseudo-nitzschia pungens* clade I (Bacillariophyceae) in the northwestern Adriatic Sea

Stefano Accoroni ^1,2,^*, Sonia Giulietti ^1^, Tiziana Romagnoli ^1^, Melania Siracusa ^2^, Simone Bacchiocchi ^2^, Cecilia Totti ^1,3^

^1^ Dipartimento di Scienze della Vita e dell'Ambiente, Università Politecnica delle Marche, via Brecce Bianche, 60131 Ancona, Italy; s.accoroni@univpm.it (S.A.); s.giulietti@pm.univpm.it (S.G.); t.romagnoli@univpm.it (T.R.); c.totti@univpm.it (C.T.)

^2^ Istituto Zooprofilattico Sperimentale Umbria e Marche, Via Cupa di Posatora, 3, 60131 Ancona, Italy; m.siracusa@izsum.it (M.S.); s.bacchiocchi@izsum.it (S.B.)

^3^ Consorzio Interuniversitario per le Scienze del Mare, CoNISMa, ULR Ancona, Ancona, Italy

***** Correspondence: s.accoroni@univpm.it; Tel.: +39-071-2204919


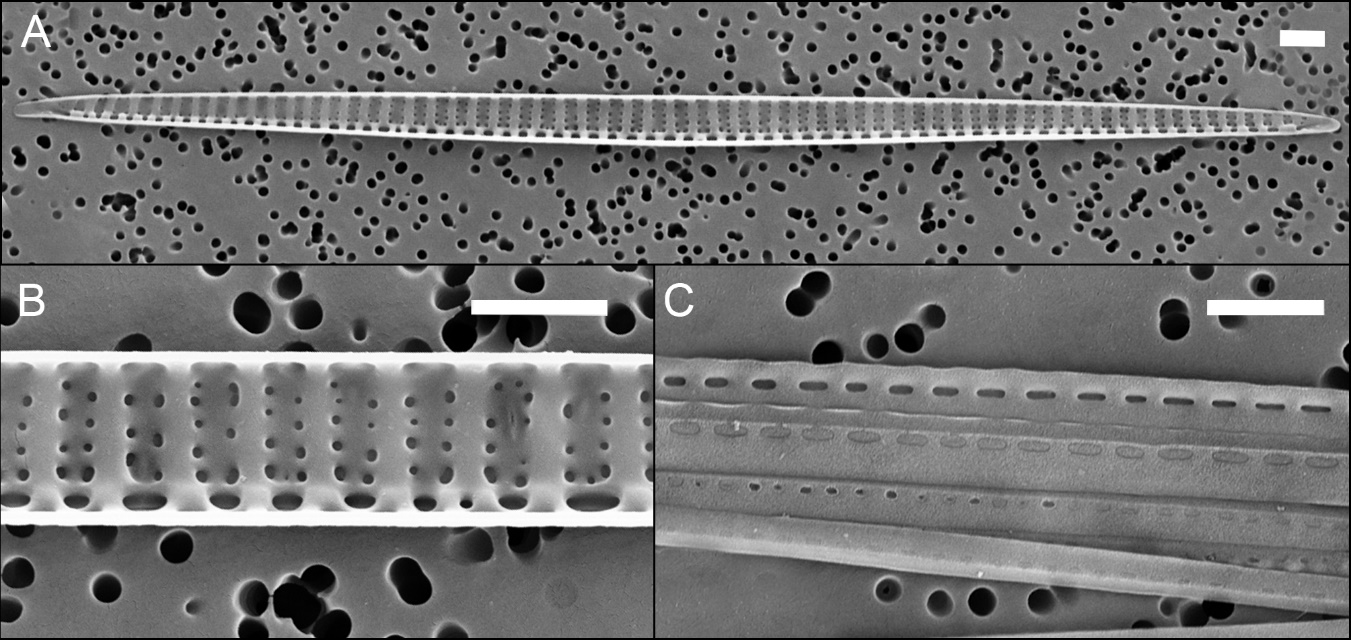


**Figure S1**. *Pseudo-nitzschia pungens* SEM micrographs (Strain ID = 01185). (**A**) Valve view. (**B**) Detail of the biseriate striae with low density of poroids. (**C**) Girdle view. Scale bar = 2 µm.


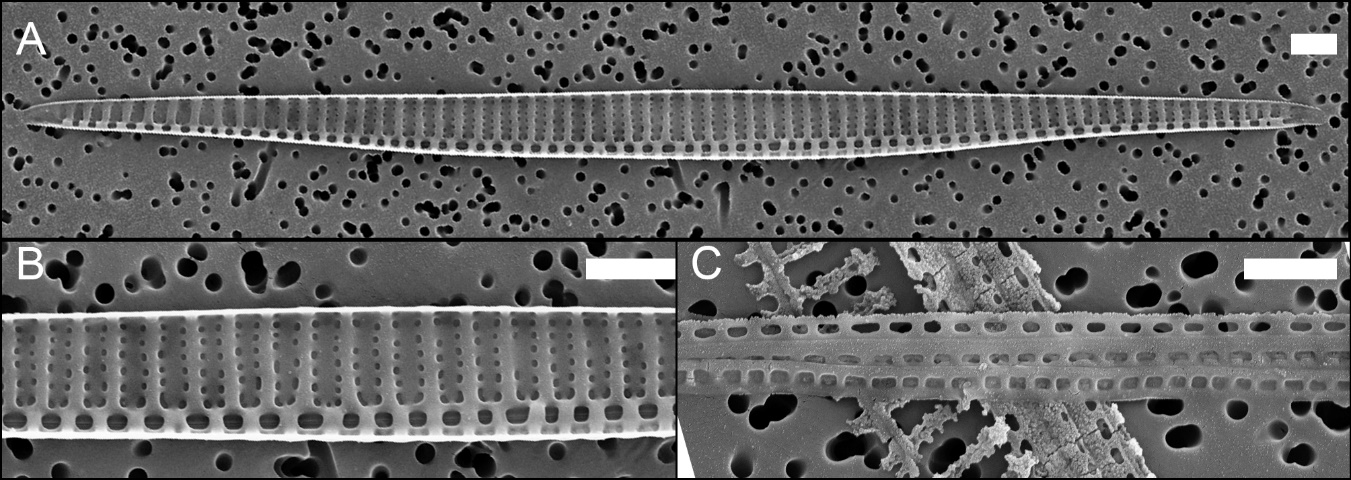


**Figure S2**. *Pseudo-nitzschia pungens* SEM micrographs (Strain ID = 01186). (**A**) Valve view. (**B**) Detail of the biseriate striae. (**C**) Girdle view: dimensions and shape of poroids in the first two cingular bands are similar. Scale bar = 2 µm.


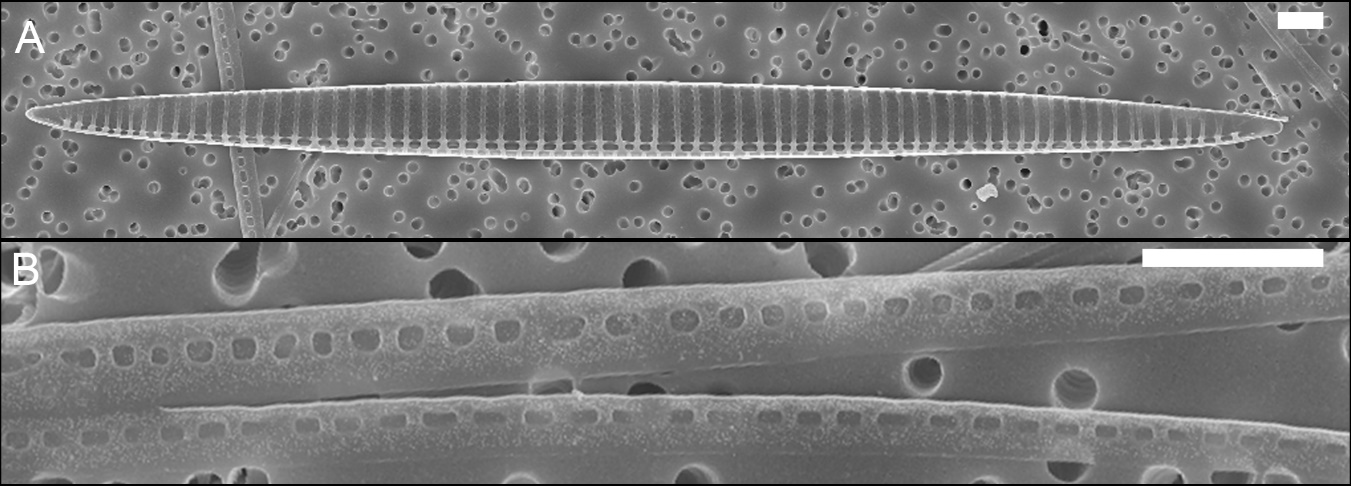


**Figure S3**. *Pseudo-nitzschia pungens* SEM micrographs (Strain ID = 01189). (A) Valve view. (B) Cingular bands showing square and rectangular poroids. Scale bar = 2 µm.

**
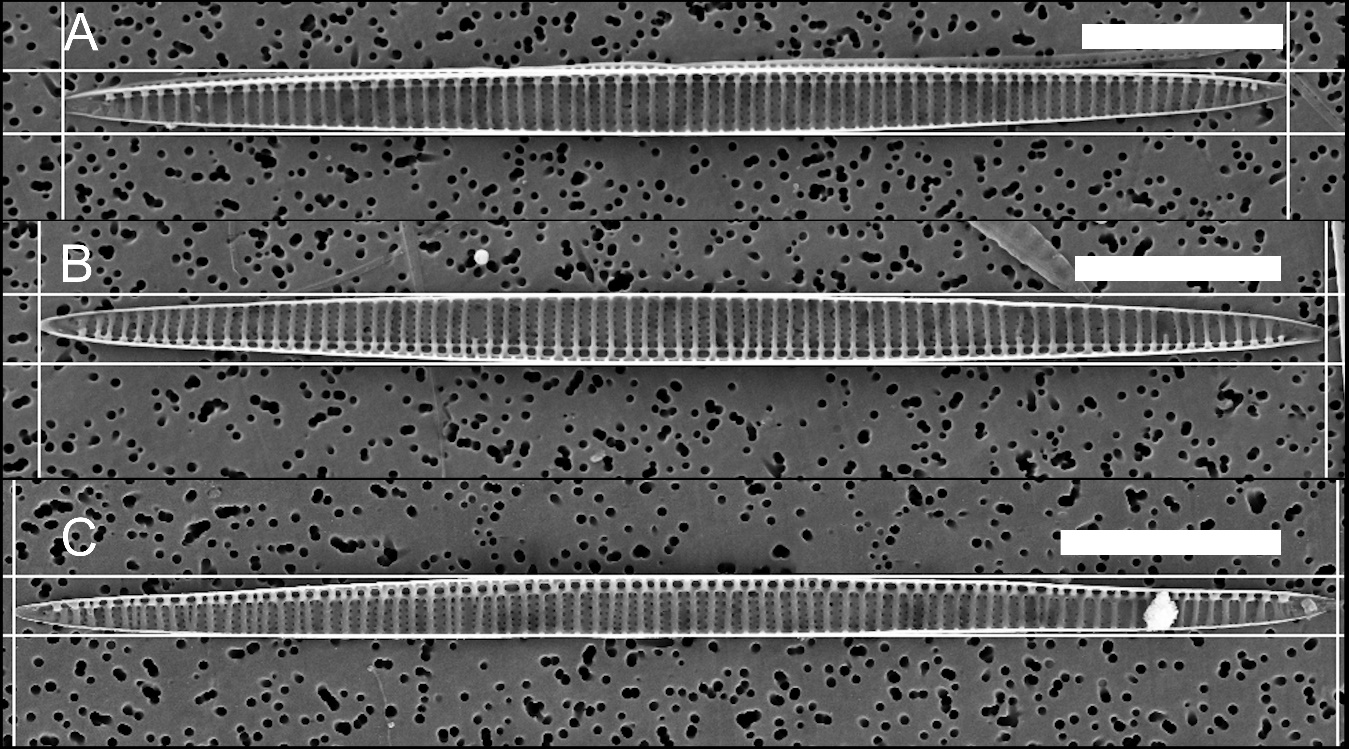
****Figure S4**. *Pseudo-nitzschia pungens* SEM micrographs (Strain ID = 031832). (**A**–**C**) Valve view showing different patterns of symmetry (highlighted by the white lines). Scale bar = 10 µm.


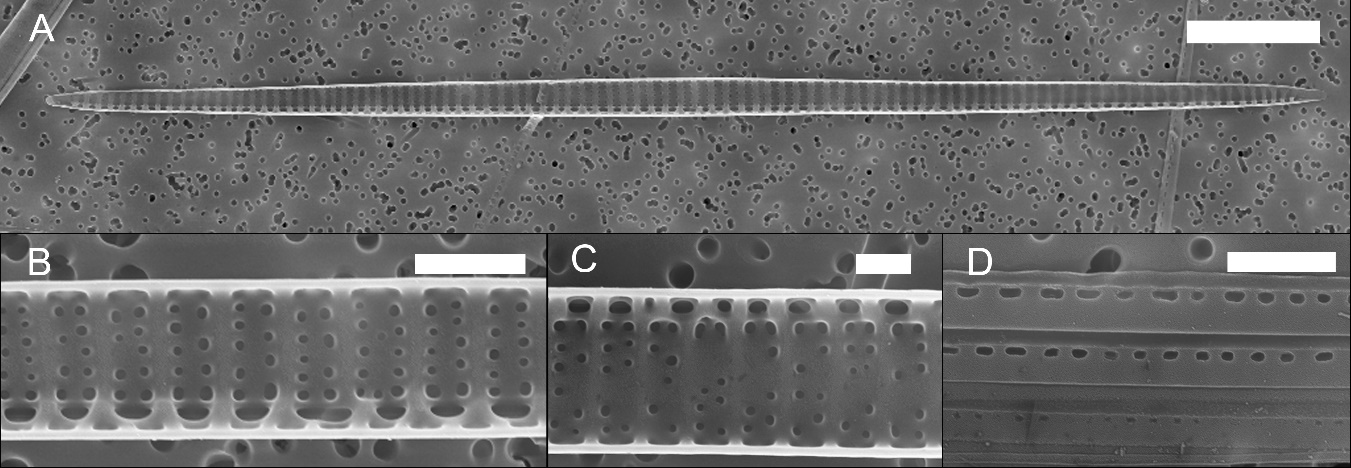


**Figure S5**. *Pseudo-nitzschia pungens* SEM micrographs (Strain ID = 04191). (**A**) Valve view. (**B**,**C**) Detail of the biseriate striae with different density of poroids. (**D**) Girdle view: dimensions and shape of poroids in the first two cingular bands are similar, while the third cingular band has smaller poroids. Scale bar = 10 µm (**A**); 2 µm (**B,D**); 1 µm (**C**).


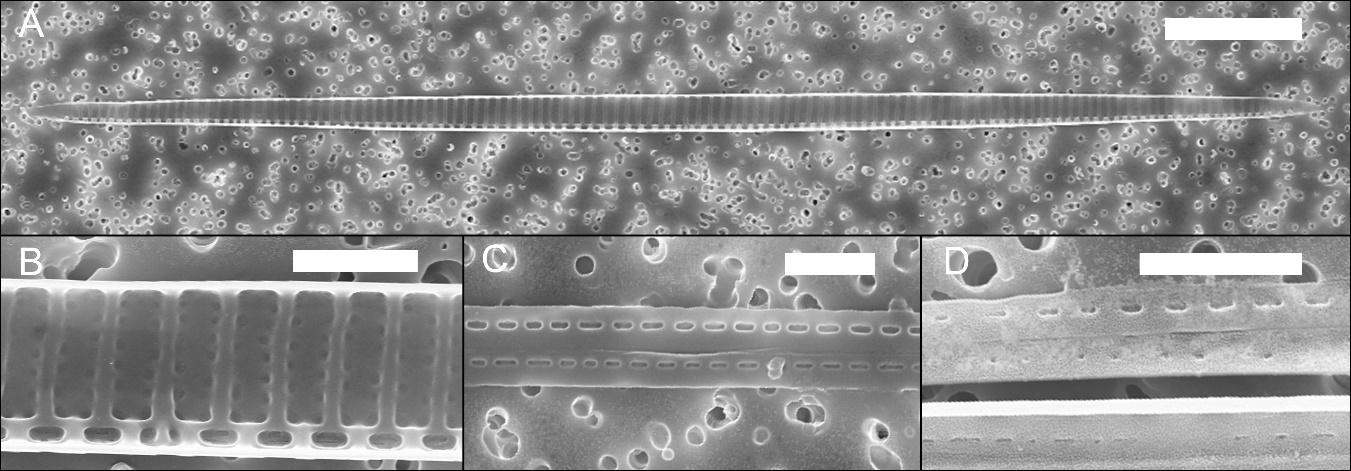


**Figure S6**. *Pseudo-nitzschia pungens* SEM micrographs (Strain ID = 04194). (**A**) Valve view. (**B**) Detail of the biseriate striae. (**C,D**) Girdle view: (**C**) dimensions and shape of poroids in the first two cingular bands are similar; (**D**) poroids with different shapes and hymenation occurring in the same cingular band. Scale bar = 10 µm (**A**); 2 µm (**B**–**D**).


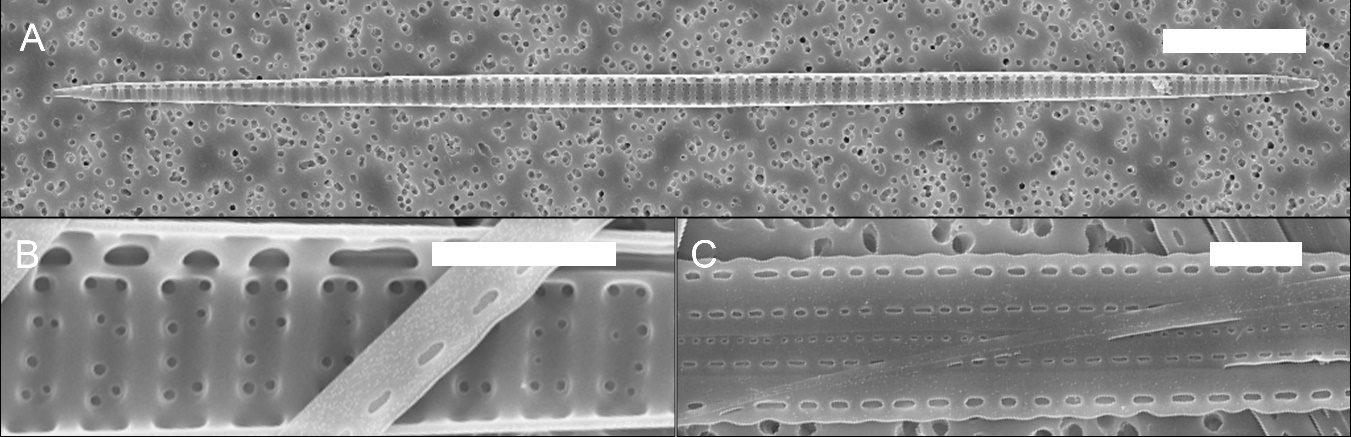


**Figure S7**. *Pseudo-nitzschia pungens* SEM micrographs (Strain ID = 04196). (**A**) Valve view of the internal valve face. (**B**) Detail of the biseriate striae with low density of poroids. (**C**) Girdle view showing a decreasing trend in abvalvar direction of the poroids’ dimensions. Scale bar = 10 µm (**A**); 2 µm (**B**–**C**).


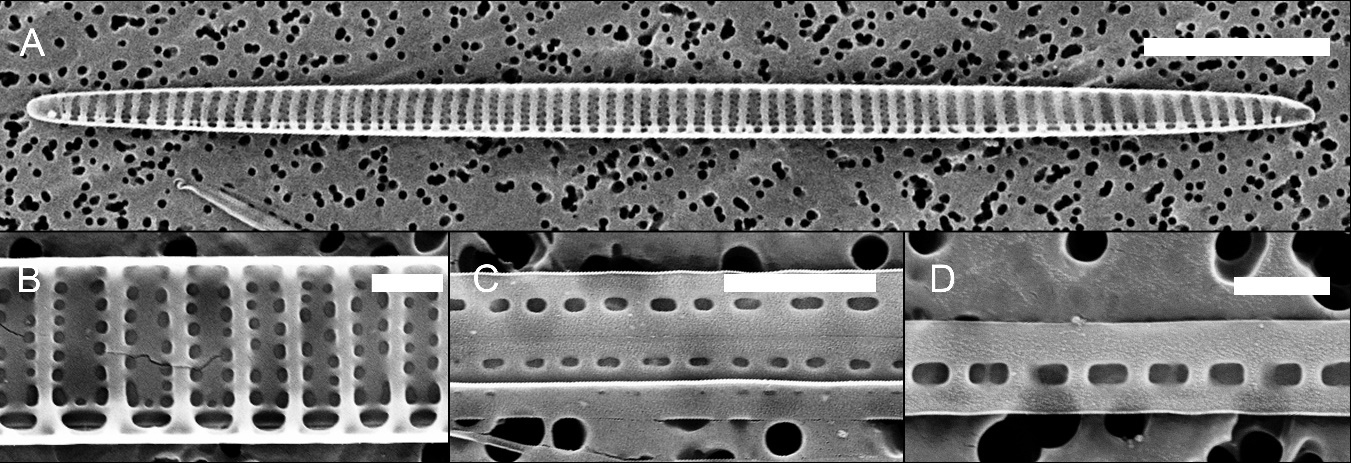


**Figure S8**. *Pseudo-nitzschia pungens* SEM micrographs (Strain ID = 05197). (**A**) Valve view. (**B**) Detail of the biseriate striae. (**C,D**) Girdle view: (**C**) dimensions and shape of poroids in the first two cingular bands are similar, while the third cingular band has smaller poroids; (**D**) poroids with different hymenation occurring in the same cingular band. Scale bar = 10 µm (**A**); 1 µm (**B,D**); 2 µm (**C**).


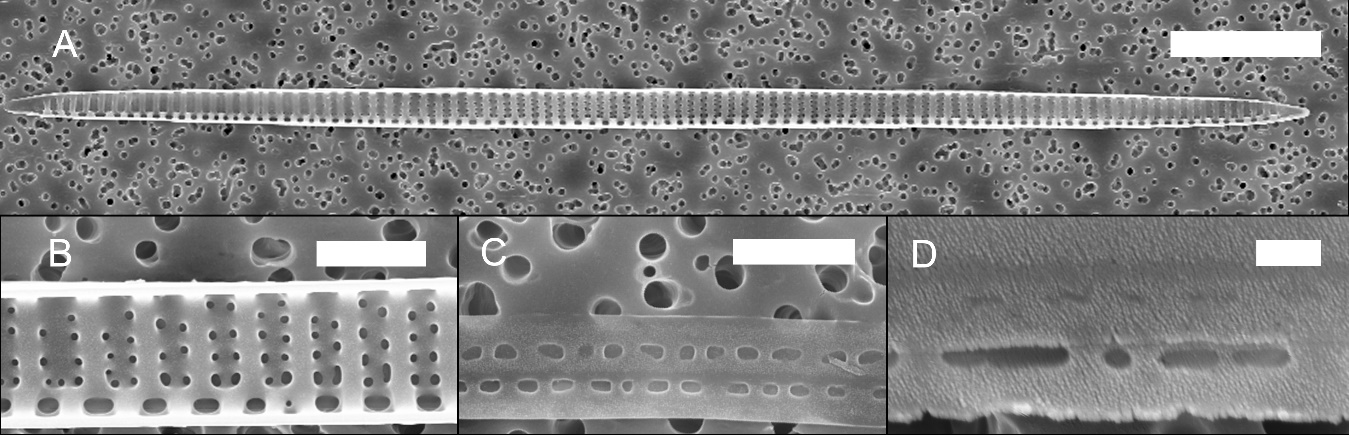


**Figure S9**. *Pseudo-nitzschia pungens* SEM micrographs (Strain ID = 05199). (**A**) Valve view. (**B**) Detail of the biseriate striae. (**C,D**) Girdle view: (**C**) dimensions and shape of poroids in the first two cingular bands are similar; (**D**) poroids with different shape and hymenation occurring in the same cingular band. Scale bar = 10 µm (**A**); 2 µm (**B,C**); 0.3 µm (**C**).


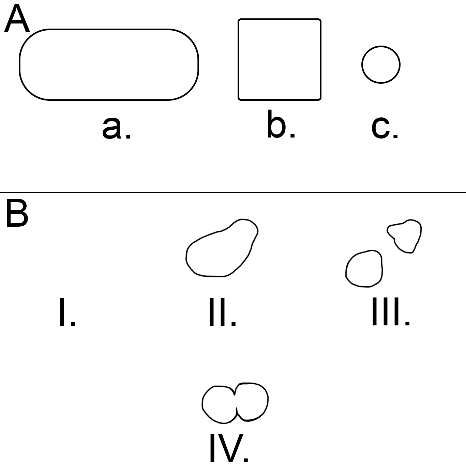


**Figure S10.** Schematic drawing describing the poroids’ morphologies of N Adriatic Sea *Pseudo-nitzschia pungens*. (**A**) Shapes of poroids: Type a: oval to rectangular. Type b: square poroids. Type c: circular. (**B**) Pattern of hymenation: Type I: no hymen sectors. Type II: one hymen sectors. Type III: two hymen sectors. Type IV: one partially divided hymen sector.


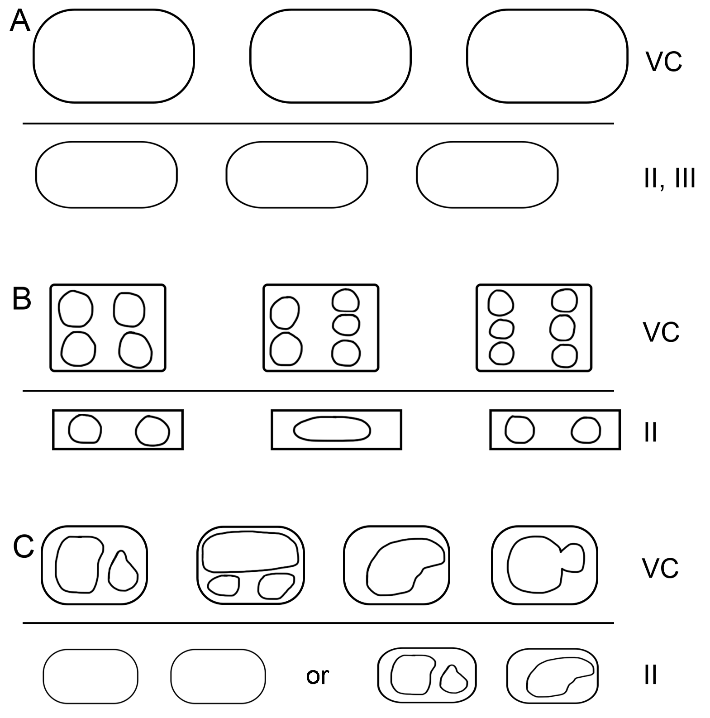


**Figure S11.** Drawing representing the ultrastructure of poroids in the girdle bands of the three *P. pungens* varieties: (A) var. *pungens* [22]; (B) var. *cingulata* [26]; (C) var. *aveirensis* [27]. VC: valvocopula; II: second cingular band; III: third cingular band. No information about the third cingular band is available in var. *cingulata* and var. *aveirensis*.


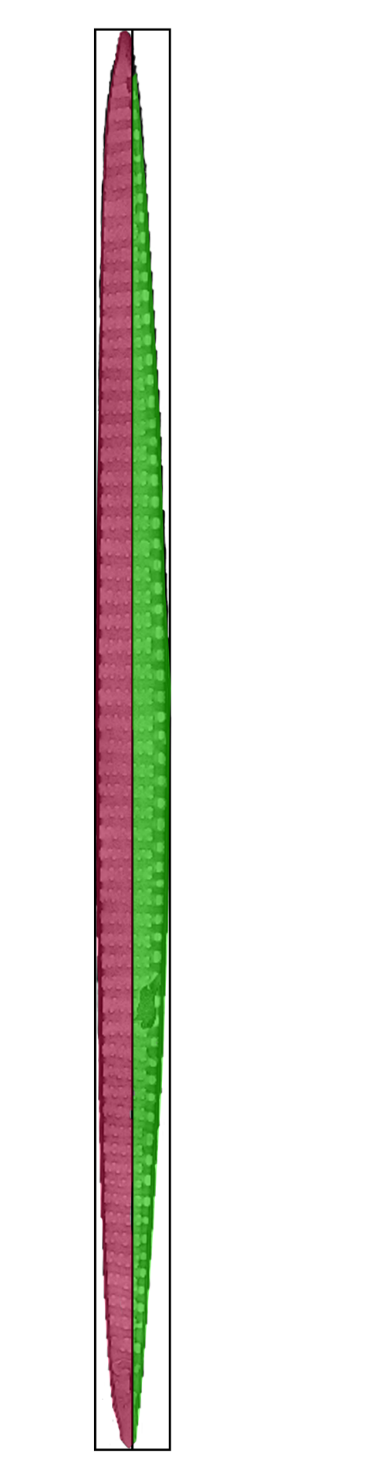


**Figure 12.** *Pseudo-nitzschia pungens* cell divided into two hemivalves. Green area: minor hemivalve; red area: major hemivalve.

**Table S1.** Morphometric characteristics of *Pseudo-nitzschia pungens* strains from the coastal site SG1 of LTER Senigallia transect. n.r., not reported.

| **Strain ID** | **Apical Axis (µm)** | **Transapical Axis (µm)** | **Fibulae in 10 µm** | **Striae in 10 µm** | **Poroids in 1 µm** | **Band Striae (in 10 µm)** | **Additional Poroids in 10 striae** |
| --- | --- | --- | --- | --- | --- | --- | --- |
| 01185 | 51.1–64.4  60.5±1.99 | 2.3–2.9  2.6±0.26 | 5–18  11.6±3.17 | 9–16  11.7±2.1 | 2–4  2.9±0.48 | 13–23  15.8±3.49 | 0–5  3.3±1.58 |
| 01186 | 56.3–63.3  59.8±3.5 | 2.1–3.3  2.8±0.27 | 8–16  11.6±1.9 | 10–13  11.5±0.87 | 2–4  3.3±0.59 | 13–19  15.2±1.6 | 0–5  2.5±1.97 |
| 01189 | 54.5-56.5  55.7±0.64 | 2.9–3.6  3.3±0.2 | 11–14  11.9±1.08 | 10–13  11.5±1 | 2–4  3±0.7 | 18–22  19.3±1.38 | 0–4  2.8±0.83 |
| 031832 | n.r. | 2.5–3.6  3.1±0.32 | 10–16  12.6±1.58 | 10–15  12±1.31 | 3–4  3.3±0.49 | 14–22  17.3±3.04 | 0–8  2.5±2.26 |
| 04191 | 93.2–96.6  94.1±1.11 | 2.2–3.1  2.8±0.31 | 10–13  10.8±1.04 | 10–11  10.6±0.52 | 2–3  2.7±0.5 | 12–20  16.2±2.58 | 0–12  3.5±4.44 |
| 04194 | 94.8–99.4  96.6±1.8 | 2.7–3.1  2.9±0.21 | 11–16  13±2 | 10–12  11.2±0.84 | 3–4  3.5±0.71 | 13–18  14.7±1.86 | 0–2 |
| 04196 | 82.9–89.5  85.8±2.37 | 2.2–2.9  2.6±0.24 | 8–16  11.4±2.24 | 9–13  10.8±1.20 | 2–4  2.9±0.64 | 13–15  14±0.63 | 0–2  1.3±0.58 |
| 05197 | 70.1–72.6  71.4±0.86 | 3.0–2.6  2.9±0.15 | 10–13  11.3±1 | 10–13  10.7±1 | 1–4  2.8±0.79 | 13–21  16.8±2.33 | 0–6  2.7±2.06 |
| 05199 | 87.9–92.0  89.5±1.51 | 2.3–3.1  2.7±0.26 | 11–12  11.4±0.55 | 10-12  10.8±0.84 | 2–3  2.8±0.45 | 13–22  16.3±2.74 | 0–3  2±1.41 |

**Table S2.** List of sequences retrieved from Genbank for the construction of the Bayesian consensus tree. Strains from this study are in bold. (c) strains of which PCR products were cloned.

| **Strain ID** | **Location** | **Date** | **Genbank Accession Number** |
| --- | --- | --- | --- |
| 1 | North Sea, Westerscheldt, The Netherlands | 24/05/2002 | AM778733 |
| 2 | North Sea, Westerscheldt, The Netherlands | 24/05/2002 | AM778734 |
| 6 | North Sea, The Netherlands | 01/05/2000 | AM778735 |
| 14 | North Sea, The Netherlands | 25/04/2002 | AM778736 |
| 16 | North Sea, The Netherlands | 22/05/2002 | AM778737 |
| 18 | North Sea, The Netherlands | 22/05/2002 | AM778738 |
| 19 | North Sea, The Netherlands | 22/05/2002 | AM778739 |
| 20 | North Sea, Belgium | 30/06/2003 | AM778740 |
| 24 | North Sea, Belgium | 30/06/2003 | AM778741 |
| 28 | North Sea, Belgium | 30/06/2003 | AM778742 |
| 40s | North Sea, Belgium | 13/08/2003 | AM778743 |
| 44 | North Sea, Belgium | 13/08/2003 | AM778744 |
| 52 | North Sea, northern France | 13/09/2003 | AM778745 |
| 57 | North Sea, northern France | 13/09/2003 | AM778746 |
| V120(3)5 | North Sea, Belgium | 24/03/2004 | AM778747 |
| V215(3)2 | North Sea, Belgium | 24/03/2004 | AM778748 |
| V215(7)3 | North Sea, Belgium | 19/07/2004 | AM778750 |
| V215(7)7 | North Sea, Belgium | 19/07/2004 | AM778752 |
| V215(7)7 | North Sea, Belgium | 19/07/2004 | AM778753 |
| V215(7)7 | North Sea, Belgium | 19/07/2004 | AM778754 |
| V215(7)7 | North Sea, Belgium | 19/07/2004 | AM778755 |
| V215(7)7 | North Sea, Belgium | 19/07/2004 | AM778756 |
| V215(7)7 | North Sea, Belgium | 19/07/2004 | AM778757 |
| S(4)6 | North Sea, Belgium | 07/04/2004 | AM778761 |
| S(4)6 | North Sea, Belgium | 07/04/2004 | AM778762 |
| S(4)6 | North Sea, Belgium | 07/04/2004 | AM778763 |
| S(4)6 | North Sea, Belgium | 07/04/2004 | AM778764 |
| S(4)6 | North Sea, Belgium | 07/04/2004 | AM778765 |
| S(4)6 | North Sea, Belgium | 07/04/2004 | AM778766 |
| S(4)6 | North Sea, Belgium | 07/04/2004 | AM778767 |
| S(7)1 | North Sea, Belgium | 13/07/2004 | AM778768 |
| W1(7)1 | North Sea, The Netherlands | 14/07/2004 | AM778769 |
| W1(7)6 | North Sea, The Netherlands | 14/07/2004 | AM778770 |
| W1(7)6 | North Sea, The Netherlands | 14/07/2004 | AM778771 |
| W1(7)6 | North Sea, The Netherlands | 14/07/2004 | AM778772 |
| W1(7)6 | North Sea, The Netherlands | 14/07/2004 | AM778773 |
| W1(7)6 | North Sea, The Netherlands | 14/07/2004 | AM778774 |
| W4(7)2 | North Sea, The Netherlands | 14/07/2004 | AM778775 |
| Vigo-2 | E Atlantic, Spain, Bay of Vigo | 01/04/2004 | AM778777 |
| Vigo-3 | E Atlantic, Spain, Bay of Vigo | 01/04/2004 | AM778778 |
| Vigo-3 | E Atlantic, Spain, Bay of Vigo | 01/04/2004 | AM778779 |
| Vigo-3 | E Atlantic, Spain, Bay of Vigo | 01/04/2004 | AM778780 |
| Vigo-3 | E Atlantic, Spain, Bay of Vigo | 01/04/2004 | AM778781 |
| Vigo-3 | E Atlantic, Spain, Bay of Vigo | 01/04/2004 | AM778782 |
| Vigo-3 | E Atlantic, Spain, Bay of Vigo | 01/04/2004 | AM778783 |
| Vigo-3 | E Atlantic, Spain, Bay of Vigo | 01/04/2004 | AM778784 |
| Vigo-4 | E Atlantic, Spain, Bay of Vigo | 01/04/2004 | AM778785 |
| Cn-172 | NW Atlantic, Canada, Prince Edward Island | 05/09/2002 | AM778786 |
| Cn-181 | NW Atlantic, Canada, Miramichi Bay | 23/09/2002 | AM778787 |
| Cn-193 | NW Atlantic, Canada, Bay of Fundy | 09/10/2002 | AM778788 |
| Cn-201 | NW Atlantic, Canada, Miramichi Bay | 15/09/2003 | AM778789 |
| Cn-205 | NW Atlantic, Canada, Prince Edward Island | 27/10/2003 | AM778790 |
| Cn-213 | NW Atlantic, Canada, Prince Edward Island | 07/09/2004 | AM778791 |
| Cn-215 | NW Atlantic, Canada, Prince Edward Island | 07/09/2004 | AM778792 |
| Cn-216 | NW Atlantic, Canada, Prince Edward Island | 07/09/2004 | AM778793 |
| Cn218 | NW Atlantic, Canada, Prince Edward Island | 07/09/2004 | AM778794 |
| US-77 | NE Pacific, North America, La Push | 18/09/2001 | AM778795 |
| US-93 | NE Pacific, North America, Mud Bay | 17/06/2002 | AM778796 |
| US-94(c) | NE Pacific, North America, Sequim Bay State Park | 08/08/2002 | AM778797 |
| US-94(c) | NE Pacific, North America, coastal Washington | 08/08/2002 | AM778798 |
| US-94(c) | NE Pacific, North America, coastal Washington | 08/08/2002 | AM778799 |
| US-94(c) | NE Pacific, North America, coastal Washington | 08/08/2002 | AM778800 |
| US-94(c) | NE Pacific, North America, coastal Washington | 08/08/2002 | AM778801 |
| US-96 | NE Pacific, North America, coastal Washington | 08/08/2002 | AM778802 |
| US-115 | NE Pacific, North America, coastal Washington | 08/08/2002 | AM778803 |
| US-123 (c) | NE Pacific, North America, coastal Washington | Jun-03 | AM778804 |
| US-123 (c) | NE Pacific, North America, coastal Washington | Jun-03 | AM778805 |
| US-123 (c) | NE Pacific, North America, coastal Washington | Jun-03 | AM778806 |
| US-123 (c) | NE Pacific, North America, coastal Washington | Jun-03 | AM778807 |
| US-123 (c) | NE Pacific, North America, coastal Washington | Jun-03 | AM778808 |
| US-123 (c) | NE Pacific, North America, coastal Washington | Jun-03 | AM778809 |
| US-132 | NE Pacific, North America, coastal Washington | Jun-03 | AM778810 |
| Us-135 | NE Pacific, North America, coastal Washington | Jun-03 | AM778811 |
| Jp-01 | NW Pacific, Japan, Ofunato Bay | 07/08/2000 | AM778812 |
| Jp-11 | NW Pacific, Japan, Ofunato Bay | 25/06/2001 | AM778813 |
| Jp-14 | NW Pacific, Japan, Ofunato Bay | 28/07/2000 | AM778814 |
| NZ-49 | S Pacific, New Zealand, North Island | Oct-00 | AM778815 |
| NZ-67 | S Pacific, New Zealand, Stewart Island | Feb-03 | AM778816 |
| NZ-74 | S Pacific, New Zealand, South Island | 2004 | AM778817 |
|  | E Atlantic, Portugal |  | AY257845 |
| Mex-18 | W Atlantic, Gulf of Mexico, Mexico, Near Tuxpam |  | AY257846 |
|  | W Pacific, Vietnam |  | DQ166533 |
| KBH2 | W Pacific, Vietnam |  | DQ062665 |
| CBA101 | Mediterranean, Italy, NW Adriatic Sea | 20/01/2010 | HE650959 |
| CBA106 | Mediterranean, Italy, NW Adriatic Sea | 20/01/2010 | HE650964 |
| mu3 | Monterey Bay, California, USA |  | AY257844 |
| **01185** | Mediterranean, Italy, NW Adriatic Sea, SG01 LTER | Jan-18 | MW114840 |
| **01186** | Mediterranean, Italy, NW Adriatic Sea, SG01 LTER | Jan-18 | MW114839 |
| **01188** | Mediterranean, Italy, NW Adriatic Sea, SG01 LTER | Jan-18 | MW114842 |
| **01189** | Mediterranean, Italy, NW Adriatic Sea, SG01 LTER | Jan-18 | MW114841 |

**Table S3**. List of *Pseudo-nitzschia pungens* strains from the coastal site SG1 of LTER Senigallia transect, cleaned for morphological characterization with TEM and SEM. Strain IDs indicated in bold are those analyzed for toxin content.

| **Strain ID** | **Isolation date** | **Age (months)** |
| --- | --- | --- |
| **01185** | January 2018 | 15 |
| **01186** | January 2018 | 15 |
| **01189** | January 2018 | 20 |
| **031832** | March 2018 | 15 |
| 04191 | April 2019 | 5 |
| 04194 | April 2019 | 5 |
| 04196 | April 2019 | 5 |
| 05197 | May 2019 | 5 |
| 05199 | May 2019 | 5 |
